# Developing a new justification for assent

**DOI:** 10.1186/s12910-015-0085-x

**Published:** 2016-01-12

**Authors:** Amanda Sibley, Andrew J. Pollard, Raymond Fitzpatrick, Mark Sheehan

**Affiliations:** Department of Paediatrics, University of Oxford, Oxford, UK; NIHR Oxford Biomedical Research Centre, Centre for Clinical Vaccinology and Tropical Medicine (CCVTM), Churchill Hospital, Old Road, Oxford, OX3 7LE UK; Nuffield Department of Population Health, University of Oxford, Richard Doll Building, Old Road Campus, Oxford, OX3 7LF UK; The Ethox Centre, Nuffield Department of Population Health, University of Oxford, Richard Doll Building, Old Road Campus, Oxford, OX3 7LF UK

**Keywords:** Assent, Research ethics, Children’s rights, Moral worth, Autonomy, Best interests

## Abstract

**Background:**

Current guidelines do not clearly outline when assent should be attained from paediatric research participants, nor do they detail the necessary elements of the assent process. This stems from the fact that the fundamental justification behind the concept of assent is misunderstood. In this paper, we critically assess three widespread ethical arguments used for assent: children’s rights, the best interests of the child, and respect for a child’s developing autonomy. We then outline a newly-developed two-fold justification for the assent process: respect for the parent’s pedagogical role in teaching their child to become an autonomous being and respect for the child’s moral worth.

**Discussion:**

We argue that the ethical grounding for the involvement of young children in medical decision-making does not stem from children’s rights, the principle of best interests, or respect for developing autonomy. An alternative strategy is to examine the original motivation to engage with the child. In paediatric settings there are two obligations on the researcher: an obligation to the parents who are responsible for determining when and under what circumstances the child develops his capacity for autonomy and reasoning, and an obligation to the child himself. There is an important distinction between respecting a decision and encouraging a decision. This paper illustrates that the process of assent is an important way in which respect for the child as an individual can be demonstrated, however, the value lies not in the child’s response but the fact that his views were solicited in the first place.

**Summary:**

This paper demonstrates that the common justifications for the process of assent are incomplete. Assent should be understood as playing a pedagogical role for the child, helping to teach him how specific decisions are made and therefore helping him to become a better decision-maker. How the researcher engages with the child supports his obligation to the child’s parents, yet why the researcher engages with the child stems from the child’s moral worth. Treating a child as having moral worth need not mean doing what they say but it may mean listening, considering, engaging or involving them in the decision.

## Background

In paediatric research, the enrolment process requires permission from the parents (i.e. proxy informed consent) with additional assent from the child in some cases^1^ [1]. Assent is generally defined as “a child’s affirmative agreement to participate in research” [2], however current guidelines do not clearly outline when assent should be attained, nor do they detail the necessary elements of the assent process [3, 4]. Instead, it has been left to paediatric researchers and local Research Ethics Committees to determine both when a child’s assent is required and how that assent should be taken and recorded [5]. The importance of this area of research has recently been highlighted with the publication of the Nuffield Council on Bioethics report on children and clinical research [4]. While the goal of the assent process is to involve those children who are sufficiently able to participate in the decision-making process, while excluding those who are not, there remains significant disagreement and lack of clarity about what counts as “sufficiently able” and how to properly assess which children are judged to be so. Consequently, research related to assent has attempted to justify a uniform rule for establishing which children should be asked for assent. This has led researchers to recommend various age-related cut-offs for assent [6, 7], while others have come to question the very validity or usefulness of assent [8]. Perhaps the reason that no consensus has been established stems from the fact that these conflicting views represent an attempt to provide an answer before the question itself is properly understood: namely what is the fundamental justification behind the concept of assent?

Many children and perhaps all who are under the age of 10 are not considered to be sufficiently competent to make their own decisions regarding their participation in clinical research. Instead, provision is usually made for a parent or guardian to consent to participation on behalf of the child. This method of dealing with enrolment would seem to leave little reason for involving children in the decision-making process. Yet the existence of the concept and practice of assent illustrates that the medical community is still motivated to include some children in this process even if they are not believed to be capable of making the decision. What matters here, then, is how to justify their involvement in the decision-making process. That is, there seems to be a general sense that we should involve these children, but it’s not immediately clear why this is warranted. Because it is not immediately clear why, it is also not clear how and when they should be involved. Current literature cites a number of different ethical justifications for some form of assent process, including reference to a child’s human rights [9–12] or respect for the child’s developing autonomy [3, 13, 14]. Without being able to agree on the fundamental justification for an assent process, it is not surprising that it is difficult to determine what assent is and how it should be documented.

In this paper, we start by critically assessing three of the most widespread current ethical arguments used for assent: children’s rights, the best interests of the child, and respect for a child’s developing autonomy. We then outline a newly-developed two-fold justification for the assent process: respect for the parent’s pedagogical role in teaching their child to become an autonomous being and respect for the child’s moral worth.^2^ This novel justification for assent is just that: a *justification* for the assent process. This paper is not concerned with why the original recommendations for assent were put in place or by whom. Instead, we are interested in outlining a solid ethical justification for involving a child in the decision-making process about his participation in clinical research.

It is important to note that in this paper assent is defined not as the child’s affirmative answer but rather the participation of the child in the overall decision-making process: focusing on the ongoing process rather than a specific answer [4]. Just as consent is not about getting the person to agree (or to consent) but, properly understood, is about allowing the person to decide (one way or the other), assent here refers to a process of engaging with a non-competent or partially competent individual and giving him an opportunity to have a say. In what follows, ‘assent’ will be taken to refer to a process of engaging with or involving the child in the decision-making, not simply getting the child to agree, to ‘go along’, or to cooperate. It is important not to underestimate this point. In order to remind the reader that what we mean by assent is the process of engagement, rather than the solicitation of a specific answer, we will refer to assent throughout the remainder of this paper as “assent*”. In addition, our discussion of assent* for the purposes of this paper concentrates specifically on the research context rather than the clinical context. We also will not discuss the differences between therapeutic and non-therapeutic research as the aim of the paper is to develop the primary justification for how and why we engage with the child, regardless of the type of research in question or its potential risks and benefits.

Finally, the reader should remember that this paper is a philosophical one, but as such it makes no presuppositions, negative or positive, about a child’s ability to meaningfully engage in decision-making, nor does it question the extent to which children can meaningfully participate in decision-making. In our view children, like adults, should be given the opportunity to consent to participate in research when they are autonomous. However there is a range of children who are not autonomous but for whom engagement with researchers and other adults may be meaningful. It is children in this category for whom assent* would seem to be appropriate. This paper is an attempt to understand and clarify the justification for involving these children in the decision-making process. It is true that much of western bioethics has been focused on the capacity to be autonomous as the primary marker of decision-making capacity [and so with particular conceptions of consent]. This paper presents a direct challenge to this orthodoxy by introducing a non-autonomy-based justification for understanding our obligations to children. As such, it runs counter to the prevailing analytic philosophy approach. However, in order to develop a compelling argument, one that might be persuasive to all readers, the paper begins with the orthodoxy in order to demonstrate its limits. Being clear about the justification requires asking “why should we involve children?” which makes it appear to be negative or sceptical. This is a key step in the philosophical method, but it does not mean that our position is a negative one; indeed, our conclusion is precisely the reverse. This method does not see the child as alien, nor does it dismiss the child until he reaches an adult age. The paper seeks to be clear about why children are not alien and why we should engage with them before they are adults.

## Discussion

### Children’s rights

It has widely been suggested that children are wrongly denied their lawful human rights of freedom and autonomy on the false premise that their chronological age automatically renders them incompetent [9–12]. In this argument, the age-based boundary between childhood and adulthood is viewed as arbitrary, and therefore unfair. With a sharp age cut-off, a younger individual may possess the necessary competencies for the holding of a specific right or making certain decisions while an older individual may not [12]. To assume blanket incompetence across all children and all situations overlooks the key fact that the acquisition of competence does not occur on a perfectly linear progression [15]. By assuming that all children under a certain age are incompetent, they are denied the appropriate degree of respect for their decision-making ability, and therefore they are unable to demonstrate their ability to accomplish the task. Without the opportunity to practice these newly learned skills, children are less likely to further expand and enhance those skills and competencies, thus hindering their overall developmental process [12].

Denying children the rights of freedom and autonomy would pose an ethical problem if they were denied solely on the basis of their chronological age, with no corresponding association to some other quality (that was ethically relevant). In fact, these rights are only withheld from children based on the assumption of an association between chronological age and the competencies deemed necessary for the possession of the rights [16]. The dividing line between these two stages of decision-making involvement will depend on the specific qualities the individual society deems necessary for an individual to be considered capable and mature enough to be involved in the decision-making [10]. It seems that there will be no single chronological age when all individuals possess a specific competence for the first time, thus entitling them to the possession of the corresponding right [12]. So, whatever the boundary, there will be some individuals who will be capable but not recognised as such and some which are not capable but are taken to be so. This observation misses the need for policy however. The goal of policy is to manage and guide practice, not to entirely circumscribe what is ethically warranted but to capture the majority of cases on either side without being arbitrary.

It is clear then, that the argument for an assent* process grounded in the child’s fundamental human rights will not work. Children do possess rights as human beings, but that does not imply that all children should be allowed either to determine or even to contribute to decisions about their own welfare. There are specific criteria to be met for the possession of such rights and there remains an association between chronological age and the attainment of these competency criteria. Most importantly, the possession of these rights as rights which entitle involvement need justification and, in particular a justification that is distinct from the justification associated with full adult competence. Children who are only able to assent* are by definition not able to consent. It is far from clear that the rights defence of an assent* process, unassisted by independent argument, can justify the kinds of involvement practices that are usually associated with assent*.

### Best interests

A second common ethical justification for assent* stems from the idea that a child’s best interests must be the overriding factor in any decision that will affect that child [17]. On this view, the child’s assent* acts as a way of providing evidence for what is in the child’s interests.

However, in the medical context, it is precisely a concern for the child’s best interests, both short-term and long-term, that motivates us to deny children any degree of self-determination. Ultimate medical decision-making power currently rests solely with the child’s caretakers, rather than shared with the child, until the child reaches the legal age of majority [1, 16]. The assumption here is that parents or guardians are the people best acquainted with their children and therefore they should be capable of making decisions that are truly in the best interests of their child and the child’s future adult self [18].

However, while it is hoped that the decisions made by parents on behalf of their children are in fact in the best interests of those children, there are too many examples of situations when this is not the case. On an extreme level, cases of child neglect and abuse illustrate that not all parents and guardians truly have a child’s best interests at heart. A parent or guardian could also justify a decision that he knows is against the desires of a child by merely stating that the decision had been made according to the best interests principle [19]. Beyond these more severe instances, even a well-intentioned parent will be making a decision based on his own subjective opinion of the child’s “best interests”, a concept nearly impossible to define [17]. A child’s best interests may change over time, depending on that child’s age, developmental stage, culture, and current environment. It is often suggested that one should consider the future adult that the child will become, making the decision that this future adult would make [12, 17]. This is sometimes described as “future-oriented consent”: as a child becomes a rational adult he will recognize the wisdom in those past decisions and lend his support to them [9, 16]. The problem with this suggestion, however, is that it can never be known what a child’s future adult self might think about the situation. Instead, a decision can only be made that in the current view would seem to be the best possible decision for the child at that time [16]. The decisions that parents make on behalf of their child shape the future person that child will become, thereby affecting what his best interests are going to be. If that is the case, those best interests cannot then be used as criteria in the current decision.

Acting according to the best interests principle will not allow us to provide an appropriate ethical justification for the process of assent*. We routinely take it that the parent or guardian has the authority to determine what the child’s interests are except in certain cases where various forms of medical authority take precedence.

### Developing autonomy

A more widely accepted and natural justification for assent* in the literature is related to respect for the child’s developing autonomy [3, 13, 14]. As children mature from infancy through to adulthood, their maturation is not purely physical but also mental, with the development of increasing cognitive capacities and an expanding ability for self-determination. Adults are asked for their informed consent to research participation out of respect for the fact that they are autonomous individuals and their decisions should be respected [15]. If all instances of full autonomy must be respected, it could be argued that instances of partial autonomy should also be respected [20]. It seems plausible that when a child has developed sufficient cognitive abilities to make decisions about his own interests, he should be allowed a corresponding degree of control over these interests [17, 21]. Asking a child to participate in the decision-making process allows him a voice in the discussion, to the extent that his current cognitive capacity will allow [3]. It looks reasonable, on this view, to assess the child’s current capacities in order to have an idea as to what level of decision-making contribution he can be expected to make. This participation not only respects the child’s increasing abilities, but also gives that child a safe forum in which to learn and practice decision-making.

However, the argument that the justification for the child’s involvement in the decision-making process lies in respect for his developing autonomy is problematic for several reasons. First, the attempt to describe children as “competent” or “incompetent” to assent* is misleading. The idea of assent* is relevant here precisely because children are not considered to be sufficiently competent to make their own decisions. To then introduce a second level of competence, now applied to a child’s ability to be involved in the decision-making process rather than making the entire decision himself, further complicates the issue and makes it even more likely that there will be confusion regarding which children should be involved. In fact, this terminology has generated much confusion and has led some researchers to recommend lowering the age of consent to include these “competent” minors, bypassing assent* altogether [22]. If a set of criteria is proposed by which to judge an individual’s competence to provide his own consent, anyone not meeting those criteria should be labelled as incompetent. There cannot then be a reclassification of some of these individuals as “quasi-competent” (which is essentially what “competent to assent*” means) based on a different set of criteria.

A second problem with the developing autonomy argument rests with the ideas of “partial autonomy” and “partial respect”. If instances of complete autonomy are fully respected, and instances of a complete lack of autonomy are not respected, then instances of partial autonomy would seem to justify partial respect. Yet it is unclear how “partial autonomy” and “partial respect” can be defined. Autonomy and respect are traditionally binary concepts [23]: a choice is either autonomous or non-autonomous, and something can either be respected or not respected. The process of assent* covers a broad range of capacities and this range would seem to be greater than any similar range among autonomous adults. The term “partial autonomy” poses a problem as it is not obvious how to determine whether an individual choice is autonomous and thus should be respected. Assent* represents an attempt to engage with children who exist along a sliding scale of competence found between a non-autonomous infant and a fully autonomous adult [24]. When confronted with a choice by a normally functioning adult, it is assumed that he is autonomous unless proven otherwise, and therefore his choice is also assumed to be autonomous. But these assumptions do not apply to a child’s “partial autonomy”. In this case, a child is already considered as not fully autonomous, so one interpretation of “partial autonomy” would seem to be that only a subset of the child’s choices are autonomous while the rest are not. It is then necessary to distinguish between his choices that are autonomous and those that are non-autonomous. However, if this is the case then the child’s autonomous choices should be accorded full respect precisely because they are autonomous, and therefore this would be justification for seeking his consent in these instances rather than assent*. The problem as it relates to assent* is that the developing autonomy argument has already indicated that partial autonomy warrants only “partial respect”, a notion that is no easier to grasp. “Partial respect” of a child’s decisions would seem to indicate that some of his decisions would be respected while other decisions would not be respected. This is problematic as none of the child’s decisions are being respected in the assent* process. Assent* currently allows some children to be involved in the decision-making process, yet the child’s decision is ultimately not accorded respect since a child’s decision can be overridden [25–27]. This usually occurs when the child’s decision is in conflict with the views of the clinician researcher and parents about what is medically in his best interests [27]. A child’s decision is not accepted as an authority when finalizing the clinical research procedures, leaving open the possibility that the final decision reached might be contradictory to the child’s stated preference. In this occurrence, “partial respect” would in effect have no meaning. If the child’s decision is not taken into account when making the final decision, then it is not being respected. Therefore, although a child does develop the capacity for autonomy throughout childhood, it is unclear how the idea of respecting this developing capacity can serve to justify the process of assent* primarily because the assent* process does not amount to respecting the decisions of the child – the parent or guardian in most cases provides the authoritative decision.

Some might argue that respect for developing autonomy requires that we engage with the child precisely because of his developing autonomy. Yet why should the fact that an individual’s autonomy is developing automatically mean that we should engage with him? Usually it is the possession of autonomy that is taken to be of ethical significance; that is, when the individual possesses the capacity, his decisions ought to be respected. In the case of developing autonomy, the individual does not possess the requisite capacity and so, the justification for respect is absent.

### Pedagogical role

So far this paper has examined how the ethical grounding for the involvement of young children in medical decision-making does not stem from children’s rights, the principle of best interests, or respect for their developing autonomy. An alternative strategy is to examine the original motivation to engage with the child. We might suggest that involving a child in the decision-making process about a clinical research procedure that affects him is motivated by the researcher’s obligations to the child: how the researcher ought to act towards that child. A medical researcher confronted with a potential child participant may feel that he ought to engage with the child in some way about the decision-making process. This obligation is less about something possessed by the child, such as his fundamental human rights or developing autonomy, but about the obligation of the researcher towards that child.

So what is that obligation? In order to formulate an answer to this question, the problem should be examined from the opposite perspective. Instead of searching for something in the child that grounds the obligation on the researcher to involve him in the decision, we might begin by examining the benefits that the child might receive if he were to be involved in the decision-making process. One obvious benefit for the child would be that his involvement would teach him how to become a better decision-maker.^3^ The pedagogical move is a very familiar one – in order to learn how to perform a task properly, the student must be given an opportunity to try and to fail [but in a protected way]. So in the case of decision-making the child is learning how to make decisions by practicing them and sometimes, perhaps often, making mistakes. By allowing the child to participate in the decision, without placing the onus on the child to make the entire decision, he is given the opportunity to practice decision-making while still under the protection of his caretakers and other adults such as doctors and nurses. Here, the importance lies in the appropriate engagement with a child, rather than on soliciting a finite “yes” or “no” response from the child. When the engagement is done poorly, either by the parent or the researcher, a child certainly might become disillusioned if he believes that his decisions are not being followed. However, this does not mean that appropriate engagement with children cannot be a very positive learning experience. The process of assent* utilizes his developing capacities and gives him a voice in matters which affect him, but most importantly it allows him to practice his decision-making skills in order to further enhance his development and become a better decision-maker as an adult [13, 24, 28, 29].

The key difference between this argument and one grounded in developing autonomy is the important distinction between respecting a decision and encouraging a decision. It is true that a child is developing increasing cognitive capacities throughout childhood, thereby forming improved decision-making abilities [20, 30]. This decision-making should be encouraged in order to further the child’s development [21, 30], but that encouragement does not need to be justified by respect. Instead, encouraging the child’s newly developed decision-making capacities is based upon the aim of teaching him, and not, in fact, respecting him.

Yet on further analysis, while assent* does teach children to become better decision-makers, this pedagogical role does not completely justify the act of involving a child in the decision-making process. If the sole concern were to teach children to make decisions, then the main focus of the obligation would be one that assists the parent rather than the child. Parents are ultimately responsible for this pedagogical role, determining throughout a child’s upbringing when and under what circumstances the child develops his capacity for autonomy and reasoning [3, 29, 31]. It is up to the parents to determine where the child falls on the spectrum of decision-making development at any given time. A good parent,^4^ who is successful in rearing his child to become an autonomous adult, will teach the child to adopt a perspective towards himself that is very like an ideal parental perspective [31]. This in effect means that the child will learn how to make good decisions based on the example the parent has set when making decisions for and on behalf of the child while under parental care. By adopting this ideal parental perspective as his own, the child will learn how to recognize what really is in his best interests, thereby learning the process of good decision-making, although not necessarily making the same decision that his parents would make. So, to be a “good parent”, one has the obligation to teach one’s children to be autonomous. In the context of medical decision-making, the researcher has the obligation to respect this parental obligation by allowing the parents to teach their child. Assent*, then, could be viewed as another pedagogical tool that parents can adopt in the overall education and nurturing of their child.^5^ In this respect, the researcher’s role is to assist and facilitate the kind of learning and perhaps the means of learning that the parents have adopted. Given that the parents are responsible for constructing the context in which a child develops his autonomy, this then implies that the parents should be allowed to determine whether or not their child should be included in the decision-making process, putting the onus on the parents rather than the investigator. This means that the researcher would engage with the child only in such a way that fits in with the individual pedagogical techniques of the child’s parents, thereby customizing his interaction and involvement with the child depending on how the child’s parents would like him to do so.^6^

However, as noted above, when seeking a child’s involvement in the medical decision-making process, it looks as though the researcher’s natural focus is primarily with the child rather than with his parents. This illustrates that, in addition to his obligations to the parents, the researcher also has an obligation to the child. This is seen most clearly in a situation where a researcher believes the child should be involved in the decision-making process but the parents do not agree (or vice versa). If assent* were purely justified by its pedagogical role and absent any clear issue of the child’s interests, the researcher would be content to accept the parental view that the child is not yet capable of participating in the decision. The judgment that a particular child ought to be involved, even against the opinion of his parental caretaker, demonstrates that the researcher believes that some involvement or assent* is ethically important because he feels that he has a duty specifically to the child, not the parents.

This realization is the key in completing the justification for assent*. In paediatric settings there are two obligations on the researcher: an obligation to the parents who lead the pedagogical role for their individual child, and an obligation to the child himself. These two obligations are most clearly seen when they conflict: when the researcher feels that the child should participate in the decision, while the child’s parents disagree. This conflict illustrates that the pedagogical role is not sufficient as the only justification for assent*. If it were, then the researcher would be able to pass off to the parents any involvement with the child, providing them with all of the information and asking them to involve their child to the degree that they see fit. However this would not absolve the researcher of his own obligation to the child. Therefore it is clear that there must be an additional element to the justification for assent*: one that addresses this second obligation of the researcher.

### Moral worth

Although the researcher has an obligation to the child, independent of his obligation to the parents, the grounds of this obligation are not clear. Some philosophers have argued that autonomous individuals have moral worth^7^ because they are autonomous [32]. Following this logic, a non-autonomous child cannot have moral worth. However by asking a child for assent* he is being treated as having moral worth [13]. While a child is not autonomous, he is not equated with an inanimate object or a non-autonomous being with whom all communication is impossible. Instead the inherent value of the child is recognized, illustrating that he warrants treatment as a being of worth. This recognition of the child’s inherent value and worth can play an important role in justifying the process of assent*. The child ought to be involved in the decision-making process about his own life rather than let adults make all of those decisions for him with no engagement from the child. Furthermore, a child will come to understand he is of value by being treated as if he is of value. Past research in paediatrics has indicated that children feel appreciated and valued when they are involved in some capacity in the medical decision-making process [33, 34]. This involvement is a sign of respect [35, 36] and teaches the child his inherent value and worth as an individual, a notion that will affect not just his ability to make good decisions but also every other aspect of his life as an autonomous being [37]. In fact, the development of a sense of moral worth is a fundamental step in a child’s overall maturation from child to adult.^8^ Without this sense, an individual is likely to struggle to have a happy and normally-functioning life. In the medical context, this sense of worth has been documented as having an effect on the emotional status of the child patient. Children have reported feelings of distress and anger when they were excluded from the decision-making process regarding their medical treatment, while children who were included in that process have experienced decreased nervousness or anxiety [33, 34, 38–40]. The process of assent*, or involving the child in the overall decision-making process, therefore, is an important way in which respect for the child as an individual can be demonstrated. The value lies not in the response given by the child but the fact that his views were solicited in the first place.

One might ask how this argument relates to a flat refusal by a child to participate in research (in other words, his dissent) in a case where that child lacks autonomy. If we think that the child’s refusal should be respected, by definition, it cannot be because we ought to respect the child’s autonomy. There must be some other account – moral worth. However, we might also think that the child’s parents have an important role here. If the parents are strongly committed to the child participating in the research, we can imagine cases where we may think it appropriate to continue to try to persuade or convince the child to do so. There are many ways of doing this depending on the age and development of the child, and often parents are very well equipped with such strategies, but all of these would require that the moral worth of the child be considered.

## Conclusions

The need for appropriate approaches to assent* in children has recently been restated and emphasized [4]. This paper has demonstrated that some of the common justifications for the process of assent* found in the literature are incomplete and do not, in fact, provide a full justification for the assent* process as an element of the overall enrolment process in paediatric research. Instead, assent* should be understood as playing a pedagogical role for the child, helping to teach him how the specific decisions are made and therefore helping him to become a better decision-maker in the future. Here, the researcher is working with the parents to help teach their child to be autonomous, fulfilling an obligation to the parents who lead this pedagogical role in the context of their family. Of course, the researcher must also recognize a potentially competing obligation to the child, regardless of the parents’ pedagogical techniques. How the researcher engages with the child supports his obligation to the child’s parents, yet why the researcher engages with the child stems from the child’s moral worth. By involving the child in the decision-making process, he is being treated as having moral worth and therefore learns that he is a being of moral worth. Part of the importance of the distinction between respecting autonomy and respecting moral worth is that it is not necessarily linked to the actual decision-making authority. Treating children as being valuable in themselves (as having moral worth) need not mean doing what they say but it may mean listening, considering, engaging or involving them in the decision. This is a new justification for assent* than has been seen previously in the literature precisely because it moves away from the tendency to tie the researcher’s obligation to engage with the child with the capacities of that child. The moral worth of the child gives the researcher an important ethical reason to treat that child with respect. One way of treating the child with respect is to involve him in the decision-making process to an extent that is appropriate to his ability to engage. Therefore, the focus of the researcher’s obligation does not stem from the child’s ability but from his moral worth. The child’s abilities and other contextual features help to shape the way in which the engagement proceeds. Once again, it is important to note that this paper has outlined a novel *justification* for the process of assent*. The novelty lies in the justification itself, not that children have moral worth or the fact that assent* is often obtained within current paediatric clinical research. This is a claim about the justification of the assent* process, and therefore it holds even if others have acknowledged, either in practice or in print, that children have moral worth.

Based on this ethical foundation for the concept of assent*, we can begin to move forward with the development of an appropriate logistical model for assent*. Given that one of the key elements of the justification of assent* stems from the parents’ pedagogical obligation to their child, then at the very least the determination of a child’s ability to participate in the assent* process should include an understanding of the child’s family context and degree of involvement in other kinds of decisions. Further research on assent* should focus not on the search for an ideal age or competence level for assent*, but instead on how a child’s family context can be considered and included in the overall assessment of how and when to involve that child in the decision-making process. Furthermore, while the arguments presented in this paper focused specifically on the process of assent* within the context of paediatric research, the conclusions drawn regarding the ethical justification for the assent* process can and should apply to all paediatric contexts – both research and clinical.

## Footnotes

1 This practice varies in different parts of the world. In this paper we are addressing only those countries where assent is an existing practice. This paper does not address international differences in how the law defines competence, but instead any discussion of competence refers to the philosophical or ethical definition of competence.

2 The process of philosophical argumentation involved in this paper does not claim that acknowledging a child’s moral worth is a new concept. What has not been done in the field of ethics and philosophical ethics, and what this paper does, is to connect the requirement that we get assent with the idea of the child’s moral worth. It is this connection, this justification of assent, that is the distinctive element of this paper. The distinctions drawn in this paper are distinctions about the way in which we justify the involvement of children who lack capacity in decisions about their participation in clinical research. It does not question whether children have moral worth, nor claim that this suggestion is new, but uses this idea in a novel way to justify their involvement in the decision-making process.

3 This argument is distinct from one based on a child’s best interests. The reason that we teach a child might not make reference to a child’s interests at all, even though it coincides with them. If we argue, for example, that the child has certain rights (one of which is to be taught to be a decision-maker) then our reason will not be paternalistic but will be about maintaining these rights. The key issue, and the one with which we are concerned in this paper, involves the justification of particular actions. Involving a child because it is best for him is just a different reason for involvement from involving him in order to teach him to decide. The way to see this difference is to observe cases where they pull apart – where the teaching is a right and done because of this, or where, in fact, learning to decide is not overall best.

4 A “good parent” here is defined as one who cares for the health and well being of the child and therefore does not abuse or neglect his child.

5 It is useful to be clear about the distinction between the pedagogical obligation and respecting the parents’ autonomy on how to raise their child. Parents are charged with the task of raising their child. In this respect they have an obligation to teach the child to be a decision-maker (among other things). In this paper we suggest that the researcher, in encouraging the child to ‘practice’ decision-making in the research context, assists in this obligation. However this is assistance in discharging a parental obligation and is distinct from the autonomy that we give parents to decide how this obligation is discharged. The right of the parents to decide how to raise their child certainly ought to be respected for the most part, (however there are standard cases where we limit parental freedoms). In the case of the assent* process this will amount to perhaps involving the child to the extent that the parents are comfortable or in the way they normally do in their daily lives.

6 We acknowledge that there are potentially other people involved in a child’s life besides his parents from whom he learns through engagement, however the main point for this paper is that the child’s parents have an authority and responsibility for him which other adults do not.

7 The term “moral worth” is common in philosophical usage. For the purposes of this paper, we understand moral worth to refer to the intrinsic moral value of a person, the recognition of which can morally inform our actions with respect to that individual. More on this definition can be found in numerous philosophical writings, including Rosati [31], Dillon [41], Vlastos [42], and Wittwer [43].

8 We recognize that children can develop attachment relationships to other people besides their parents and that these relationships are also instrumental in developing a child’s overall sense of moral worth. There is certainly a rich body of psychology literature on development theory, and more to be said about self worth and its nature, but that is beyond the remit of this paper.
